# Genetic determinants of age-related macular degeneration in Middle Eastern populations: a systematic review

**DOI:** 10.3389/fgene.2026.1776779

**Published:** 2026-05-08

**Authors:** Naif S. Sannan

**Affiliations:** 1 Department of Clinical Laboratory Sciences, College of Applied Medical Sciences, King Saud bin Abdulaziz University for Health Sciences, Jeddah, Saudi Arabia; 2 Biomedical Research Department, King Abdullah International Medical Research Center, Jeddah, Saudi Arabia

**Keywords:** age-related macular degeneration, AMD, ARMS2, CFH, genetics, HTRA1, middle east, polymorphism

## Abstract

**Background:**

Age-related macular degeneration (AMD) is a leading cause of vision loss, with genetic factors playing a key role in disease susceptibility and progression. While extensive genetic research is being conducted, the genetic architecture of AMD in Middle Eastern populations remains understudied. This systematic review summarizes current evidence on genetic variants associated with AMD in Middle Eastern populations.

**Methods:**

A comprehensive literature search was conducted in PubMed, Web of Science Core Collection, and Medline databases. Studies were included if they: (1) examined cohorts from Middle Eastern participants; (2) with clinically diagnosed AMD; (3) explored genetic variants or other genomic markers; (4) no restrictions on year of publication; and (5) were published in English.

**Results:**

The search yielded 449 articles (PubMed: 164, Web of Science: 99, Medline: 186). After removal of 221 duplicates, 228 unique articles were screened. Of these, 28 studies met the inclusion criteria, covering a total of 4,247 AMD cases and 3,447 controls from five countries: Turkey (n = 11), Iran (n = 11), Israel (n = 4), Jordan (n = 1), and Egypt (n = 1). Most analyses were targeted, with 25 studies targeting one to four genetic loci, two studies examining 12 variants, and one genome-wide association study. The most frequently studied genes were CFH, ARMS2, and HTRA1. The CFH Y402H variant (rs1061170) showed overall positive but heterogeneous associations with AMD risk across studied Middle Eastern populations, with reported odds ratios ranging from 0.36 to 17.34 and statistically significant p values ranging from <0.001 to 0.02 (total AMD cases and controls = 2,079). The ARMS2 A69 S variant (rs10490924) and HTRA1 promoter variant (rs11200638) demonstrated strong associations with neovascular AMD.

**Conclusion:**

Few studies have examined genotype-phenotype correlations across this region, and many Middle Eastern countries lack published AMD genetic data. Consequently, the genetic landscape of AMD in the Middle East remains incompletely characterized. Available evidence suggests that variants in CFH, ARMS2, and HTRA1 are important AMD-associated loci in studied Middle Eastern populations, consistent with findings in other population groups.

## 1 Introduction

Age-related macular degeneration (AMD) is a progressive neurodegenerative disease affecting the macula, the central region of the retina responsible for high-acuity vision. AMD poses a significant public health challenge as a leading cause of irreversible blindness in people over 60 years of age in developed countries. The global prevalence of AMD is projected to increase from 196 million in 2020 to 288 million by 2040, driven by population aging (Wong et al., 2014; Bourne et al., 2021).

AMD is a complex multifactorial disease with strong genetic contribution. Twin studies have demonstrated heritability estimates as high as 70% (Seddon et al., 2005). Genome-wide association studies (GWAS) have identified multiple genetic loci associated with AMD, with the complement pathway emerging as a central biological mechanism (Fritsche et al., 2016).

Middle Eastern populations remain significantly underrepresented in genetic research. These populations exhibit unique genetic characteristics shaped by historical migrations, founder effects, and consanguinity patterns (Bhattacharya et al., 2023; Ramaswamy et al., 2022). Understanding the genetic determinants of AMD in these populations is essential for developing population-tailored risk prediction models and to better characterize the genetic architecture of AMD.

This review aims to: (1) systematically identify all studies investigating genetic variants associated with AMD in Middle Eastern populations, (2) summarize the genetic variants and genes most consistently associated with AMD in this region, (3) identify knowledge gaps to guide future research.

## 2 Methods

The research question was defined using the PICOS framework. The population (P) included Middle Eastern individuals with clinically diagnosed AMD. The exposure (I) was genetic variation, including single-nucleotide polymorphisms, rare variants, and other genomic markers. The comparators (C) were non-AMD controls. The outcomes (O) were associations with AMD risk or subtype (dry or wet AMD). The study designs (S) included observational genetic studies, such as case-control, cohort, cross-sectional, GWAS, and candidate-gene studies. The systematic review was conducted in accordance with the Preferred Reporting Items for Systematic Reviews and Meta-Analyses (PRISMA) guidelines (Page et al., 2021).

The review included: (1) studies conducted in populations from Middle Eastern countries (Saudi Arabia, the United Arab Emirates, Qatar, Kuwait, Bahrain, Oman, Yemen, Iraq, Syria, Jordan, Lebanon, Cyprus, Palestine, Egypt, Turkey, Iran, and Israel); (2) studies enrolling participants with a clinical diagnosis of AMD; and (3) studies investigating genetic variants, polymorphisms, or other genomic markers. All original study designs were eligible, including case-control, cohort, cross-sectional, GWAS, and candidate-gene studies, with (4) no restrictions on year of publication and (5) publication in the English language. Studies were excluded if they were: (1) conference abstracts without accessible full text; (2) focused on juvenile or non-AMD disorders; (3) did not conduct genetic analyses; or (4) published in languages other than English.

A literature search was conducted in PubMed, Web of Science Core Collection, and Medline using a predefined search strategy. The strategy was developed using a combination of three key concept domains: Macular degeneration, genetics, and Middle Eastern people. Controlled vocabulary terms (e.g., MeSH) were applied in PubMed/MEDLINE, while for Web of Science, a keyword-based search using topic field tags (TS =) and Boolean operators was implemented. The full search strategy is provided in Supplementary Table S1. This review analyzed articles published and available up to the end of November 2025.

Screening of titles and abstracts was conducted, followed by full-text assessment for eligibility and data extraction from eligible studies. All steps were performed by a single reviewer using predefined criteria. The following items were extracted: study design, population, sample size (AMD types and controls), genotyping methods, genes and variants investigated, genetic associations (odds ratios, confidence intervals, p-values), and key findings. The quality of the included studies was assessed using the Newcastle-Ottawa Scale for non-randomised studies (Wells et al., 2000). This systematic review was not prospectively registered in PROSPERO.

## 3 Results

### 3.1 Study selection

**FIGURE 1:**
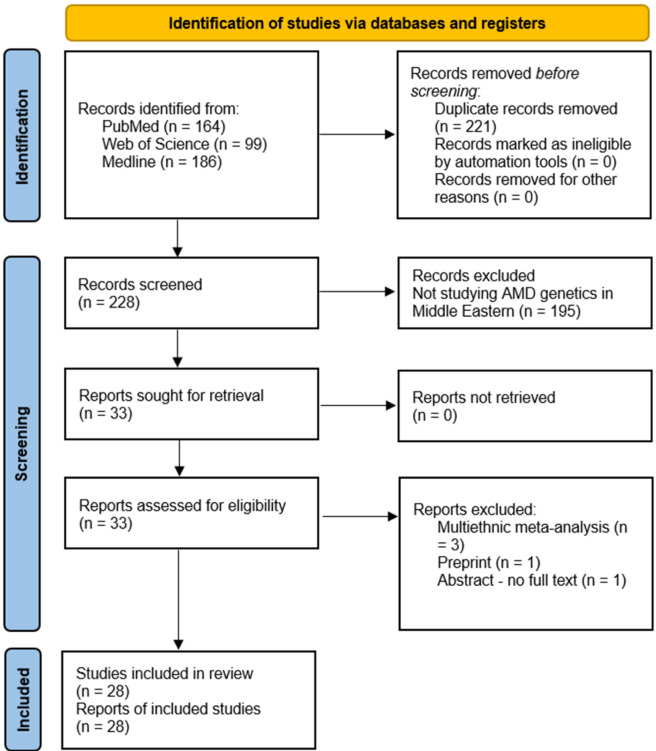
PRISMA diagram of study selection process.

The literature search identified 449 records, of which 228 unique articles remained after duplicate removal. Titles and abstracts of the analyzed articles are presented in Supplementary Table S2. A total of 195 articles were excluded after title and abstract screening for the following reasons: studies on conditions other than AMD (majority), non-genetic studies (e.g., prevalence or treatment-focused), and studies outside the scope (e.g., animal, laboratory, or non-clinical research). An additional five articles were excluded, including three multi-ethnic meta-analyses, one preprint, and one conference abstract with no available full text. Consequently, 28 studies met the inclusion criteria for this review (Table 1 (Wells et al., 2000; Abbas and Azzazy, 2013; Bonyadi et al., 2017a; Babanejad et al., 2016; Karkhane et al., 2016; Chowers et al., 2008a; Chowers et al., 2008b; Asleh et al., 2009; Furundaoturan et al., 2025; Alkhateeb et al., 2012; Üçer et al., 2011; Yücel et al., 2012; Grunin et al., 2024; Bonyadi et al., 2017b; Soysal et al., 2012; Bezci Aygun et al., 2020; Güven et al., 2011; Bulgu et al., 2014; Kepez Yildiz et al., 2016; Bardak et al., 2017; Okur et al., 2015; Çelebiler et al., 2013; Bonyadi et al., 2019; Roshanipour et al., 2019; Othman et al., 2012; Khanamiri et al., 2014; Bonyadi et al., 2015; Askari et al., 2015)). Of the 28 included studies, 24 were classified as good quality, 3 as fair quality, and 1 was not applicable for assessment using the Newcastle-Ottawa Scale due to its non-comparative case series design; however, it was included as it provided relevant data on genetic variant frequencies and phenotypic associations in AMD, contributing to the overall evidence synthesis. The study selection process is illustrated in Figure 1.

**TABLE 1:** Summary of included studies examining genetic determinants of AMD in Middle Eastern populations.

References	Cohort	Study design	Dry AMD (n)	Wet AMD (n)	AMD type not specified (n)	Controls (n)	Total sample size	Genotyping method	Gene	Protein	dbSNP rsID	Alleles or genotypes	OR (95% CI)	p-value	Key findings	Newcastle-ottawa quality scale
Abbas and Azzazy (2013)	Egyptian	Case-control	1	25	0	20	46	PCR-RFLP + Sanger sequencing	CFH ARMS2 HTRA1	Y402H A69 S non-coding	rs1061170 rs10490924 rs11200638	T/CG/TG/A	5.5 (1.7–17.3) 5.6 (2.01–15.9) 3.8 (1.2–11.8)	0.00214 0.00056 0.01357	CFH (rs1061170), ARMS2 (rs10490924), and HTRA1 (rs11200638) are significantly associated with AMD.	Good
Bonyadi et al. (2017a)	Iranian	Case-control	79	187	0	228	494	PCR-RFLP + sequencing	C3	R102G	rs2230199	C/G	1.9 (1.3–2.6)	0.0001	C3 (rs2230199) is significantly associated with advanced AMD.	Good
Bonyadi et al. (2017b)	Iranian	Case-control	76	203	0	221	500	Sequenom MassARRAY iPLEX	SMUG1	non-coding	rs3087404	A/G	1.03 (0.8–1.3)	0.83	SMUG1 polymorphism shows no association with AMD.	Good
Bonyadi et al. (2019)	Iranian	Case-control	17	203	0	151	371	PCR-RFL + Sequencing	CFI	G119R	rs141853578	C/T	1.5 (1.07–2.1)	0.018	CFI (rs10033900) is significantly associated with AMD.	Good
Roshanipour et al. (2019)	Iranian	Case-control	42	244	0	194	480	PCR-RFLP + Random Sequencing	CFB	L9H	rs4151667	AA+ AT	0.4 (0.1–1.2)	0.038	CFB (rs4151667) shows a significant association with AMD.	Good
Othman et al. (2012)	Iranian	Case-control	0	112	0	112	224	PCR	GSTM1 GSTT1	null null	- -	- -	0.9 (0.5–1.6) 0.8 (0.4–1.5)	0.889 0.542	GSTM1 and GSTT1 (null genotypes) are not associated with overall AMD risk or combined genotypes; however, GSTM1 null genotype is linked to earlier onset of wet AMD (P = 0.008)	Good
Khanamiri et al. (2014)	Iranian	Case-control	0	0	70 (geographic atrophy, choroidal neovascularization or disciform scar)	86	156	Sanger Sequencing	CFH ARMS2	Y402H A69 S	rs1061170 rs10490924	T/CG/T	- -	0.002 0.002	CFH (rs1061170) and ARMS2 (rs10490924) are significantly associated with AMD (OR = 1.9 and 2.2, respectively)	Fair
Bonyadi et al. (2015)	Iranian	Case-control	50	0	0	73	123	PCR-RFLP	TNF-α	non-coding non-coding	rs1799964 rs1800629	T/CG/A	- -	0.86 0.67	TNF-α (rs1799964 and rs1800629) show no significant association with advanced dry AMD.	Good
Askari et al. (2015)	Iranian	Case-control	32	88	0	120	240	Sanger Sequencing	HTRA1	non-coding non-coding	rs11200638 rs2672598	G/AT/C	10.1 (4.4–23.01) 2.8 (1.5–5.3)	0.001 0.001	HTRA1 (rs11200638 and rs2672598) polymorphisms are significantly more frequent in AMD patients than controls	Good
Bonyadi et al. (2016)	Iranian	Case-control	172	94	0	229	495	PCR-RFLP + sequencing	CFH CCL2	Y402H non-coding	rs1061170 rs1024611	CT AG	2.9 (1.8–4.8) 0.8 (0.4–1.5)	<0.001 0.5	CFH (rs1061170) shows a strong association with AMD, while CCL2 (rs1024611) shows no independent association	Good
Babanejad et al. (2016)	Iranian	Case-control	10	90	0	100	200	PCR-RFLP + Sanger sequencing	CFH	I62V or V62I Y402H A473= non-coding	rs800292 rs1061170 rs2274700 rs3753395	G/AC/TG/AA/T	- - - -	0.005 <0.001 <0.001 <0.001	CFH (rs800292, rs2274700, rs3753395, and rs1061170) are significantly associated with increased AMD risk	Good
Karkhane et al. (2016)	Iranian	Non-comparative case series	0	44	0	0	44	Sequencing or Sequenom MassARRAY iPLEX	CFH HTRA1 ARMS2	non-coding non-coding I62V/V62I A307= Y402H non-coding non-coding non-coding non-coding non-coding non-coding non-coding	rs203674 rs572515 rs800292 rs1061147 rs1061170 rs2274700 rs7529589 rs12038333 rs35507625 rs10664316 rs11200638 rs2672598	C/AT/CC/AA/CC/TC/TT/CG/A del del AT A/GG/A	-	-	CFH (rs1061147) was the most common variant (100% frequency), while HTRA1 (rs2672598) was the least frequent (52.27%); all other SNPs showed frequencies ranging from 63.63% - 95.45%	Not applicable
Chowers et al. (2008a)	Israeli	Case-control	0	240	0	118	358	Sequenom MassARRAY iPLEX	CFH	Y402H	rs1061170	T/C	1.9 (1.3–2.6)	0.0002	CFH (rs1061170) variant is associated with neovascular AMD.	Good
Chowers et al. (2008b)	Israeli	Case-control	0	255	0	119	374	Sequencing + qPCR	ARMS2 HTRA1	A69 S non-coding	rs10490924 rs11200638	G/TG/A	3.1 (2.2–4.5) 2.7 (1.9–4)	<0.0001 <0.0001	ARMS2 (rs10490924) and HTRA1 (rs11200638) variants are associated with neovascular AMD.	Good
Asleh et al. (2009)	Israeli	Case-control	0	290	0	157	447	PCR-RFLP	TF HFE	S570P C282Y	rs1049296 rs1800562	C/TG/A	- -	0.47 0.1	Transferrin (rs1049296) and hemochromatosis (rs1800562) variants are not associated with an increased risk of AMD.	Good
Grunin et al. (2024)	Israeli	Case-control genome wide association	0	0	403 + 155 for validation (atrophic and neovascular)	256 + 69 for validation	883	Exome chip + Sanger	C7orf50 IGDCC4 CNTNAP4 FAM189A1	non-coding W1076L non-coding non-coding	rs12701455 rs116928937 rs1506825 rs1195500	A/G,TC/AA/C,TG/A,C,T	0.5 (0.26–94) 0.1 (0.02–0.8) 0.5 (0.3–0.96) 0.5 (0.2–0.9)	<0.0001 <0.0001 <0.0001 <0.0001	31/34 AMD loci nominally replicated (p < 0.05); CFH (p = 1.6 × 10^−9^) and ARMS2/HTRA1 (p = 3.4 × 10^−9^) top signals C7orf50 (rs12701455), IGDCC4 (rs116928937), CNTNAP4 (rs1506825), and FAM189A1 (rs1195500) were significantly associated with AMD in the discovery cohort (P < 5 × 10^−5^)	Fair
Alkhateeb et al. (2012)	Jordanian	Case-control	0	0	42 (wet and dry)	92	134	PCR-RFLP	SMOC2	non-coding	rs13208776	A/G	0.84 (0.47–1.49)	0.64	SMOC2 (rs13208776) shows no association with AMD.	Fair
Üçer et al. (2011)	Turkish	Case-control	48	30	0	68	146	PCR	ACE	non-coding	rs4646994	insertion/deletion	-	0.218	ACE (rs4646994) shows no significant association with AMD, with comparable serum ACE levels and genotype frequencies between patients and controls	Good
Yücel et al. (2012)	Turkish	Case-control	69	26	0	87	182	PCR-RFLP	CFH	Y402H	rs1061170	T/C	2.3 (1.2–4.3)	0.009	CFH (rs1061170) is associated with late AMD, with TC heterozygotes showing an increased risk compared with controls (OR = 2.32)	Good
Furundaoturan et al. (2025)	Turkish	Case-control	100	0	0	50	150	MiSeq NGS platform	ARMS2	A69 S	rs10490924	GT	3 (1.2–7.4) 2.2 (0.9–5.1)	0.008* 0.034**	The ARMS2 variant (rs10490924) is significantly associated with dry AMD, regardless of the presence (*) or absence (**) of reticular pseudodrusen when compared to controls	Good
Soysal et al. (2012)	Turkish	Case-control	25	122	0	105	252	PCR-RFLP	CFH ARMS2	Y402H A69S	rs1061170 rs10490924	T/CG/T	2.1 (1.4–3.05) 3 (2.04–4.4)	0.0001 0.0001	CFH (rs1061170) and ARMS2 (rs10490924) are significantly associated with AMD.	Good
Bezci Aygun et al. (2020)	Turkish	Case-control	46	65	0	96	207	Sanger Sequencing	CFI	non-coding E115 =	rs10033900 rs2285714	- -	1.05 (0.7–1.5) 1.2 (0.8–1.8)	0.788 0.275	CFI (rs2285714 and rs2285714) shows no association with AMD.	Good
Güven et al. (2011)	Turkish	Case-control	65	55	0	198	318	Multiplex PCR + real time PCR	GSTM1 GSTT1 GSTP1	null null I105V	- - -	null nullA/G	1.8 (1.1–2.9) 0.7 (0.4–1.3) 0.9 (0.5–1.7)	0.01 - -	GSTM1 (null genotype) is significantly associated with increased AMD risk, particularly dry AMD (OR ≈ 1.8–2.0)	Good
Bulgu et al. (2014)	Turkish	Case-control	39	43	0	80	162	Real time PCR	VEGF	non-coding non-coding non-coding	rs1413711 rs2146323 rs3025033	G/AC/AA/G	2.07 (1.3–3.3) 2.2 (1.4–3.5) -	0.072 0.058 0.616	VEGF (rs1413711, rs2146323, and rs3025033) show no association with AMD.	Good
Kepez Yildiz et al. (2016)	Turkish	Case-control	0	109	0	70	179	PCR-RFLP	CFH VEGF	Y402H non-coding non-coding	rs1061170 rs699947 rs2146323	T/CA/CA/C	0.5 (0.3–0.8) 1.01 (0.6–1.5) 0.8 (0.5–1.2)	0.011 0.94 0.37	CFH (rs1061170) shows a significant genotype difference. VEGF (rs699947 and rs2416323) show no association with AMD.	Good
Bardak et al. (2017)	Turkish	Case-control	0	39	0	250	289	MiSeq NGS platform	ARMS2	R3H R38* A69S	rs10490923 rs2736911 rs10490924	GA AC TG	0.9 (0.3–2.4) 0.79 (0.2–2.3) 8.5 (3.5–20.8)	0.86 0.68 <0.0001	ARMS2 variant (rs10490924) was significantly associated with AMD.	Good
Okur et al. (2015)	Turkish	Case-control	45	42	0	80	167	Sanger Sequencing	CFH SKIV2L MYRIP HIF1A	Y402H non-coding non-coding non-coding	rs1061170 rs429608 rs2679798 rs11549465	T/CG/AG/AC/T	2.4 (1.2–4.8) 0.7 (0.3–1.5) 0.8 (0.5–1.2) 0.7 (0.4–1.3)	0.011 - - -	CFH (rs1061170) is independently associated with increased AMD risk (OR = 2.42). No other SNPs show significant associations	Good
Çelebiler et al. (2013)	Turkish	Case-control	26	29	0	18	73	Multiplex polymerase chain reaction (Cardiovascular Disease StripAssay)	F5 F5 F2 F13A1 FGB SERPINE1 ITGB3 MTHFR MTHFR ACE APOE APOE	R506Q H1299R non-coding V34L non-coding non-coding L59P A222V E429A non-coding C130R R176C	rs6025 rs1800595 rs1799963 rs5985 rs1800790 rs1799889 rs5918 rs1801133 rs1801131 rs4646994 rs429358 rs7412	-	-	-	A Bayesian network approach found a joint contribution of SERPINE1 (rs1799889), F13A1 (rs5985), F5 (rs6025), and F2 (rs1799963) were identified, achieving a predictive accuracy of 69% in distinguishing AMD cases from controls. MTHFR (rs1801133) + SERPINE1 (rs1799889) + obesity may increase wet AMD risk	Fair

### 3.2 Study characteristics

**FIGURE 2:**
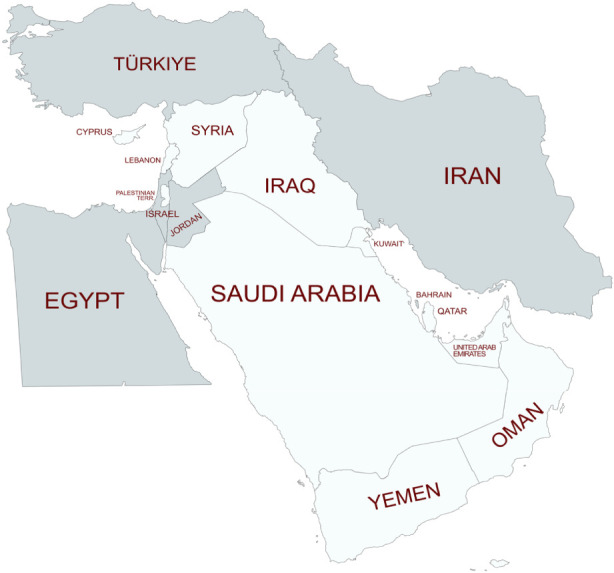
Geographic distribution of genetic studies on age-related macular degeneration (AMD) in the Middle East. Countries shown in dark grey represent those with published genetic association studies investigating AMD, while light grey countries indicate regions where no genetic studies were identified (Created with mapchart.net).

The 28 included studies comprised a total of 7,694 participants (cases and controls) from five Middle Eastern countries. The geographic distribution of studies was markedly unbalanced, with the highest representation from Iran (11 studies; 478 dry AMD, 1,265 wet AMD, 70 with geographic atrophy or choroidal neovascularization or disciform scar, 1,514 controls), Turkey (11 studies; 463 dry AMD, 560 wet AMD, 1,102 controls), and Israel (4 studies; 785 wet AMD, 558 atrophic or neovascular AMD, 719 controls), followed by Jordan (1 study; 42 participants with wet or dry AMD, 92 controls) and Egypt (1 study; 1 dry AMD, 25 wet AMD, 20 controls) (Figure 2). No eligible studies were identified from Saudi Arabia, the United Arab Emirates, Qatar, Kuwait, Bahrain, Oman, Yemen, Iraq, Syria, Lebanon, Cyprus or Palestine. Across all included studies, 942 participants had dry AMD, 2,635 had wet AMD, 670 had AMD of unspecified subtype (including geographic atrophy, choroidal neovascularization, disciform scar, wet, and dry types) and 3,447 served as controls. Most studies employed a case-control design (n = 26); one study was a non-comparative case series, and one study used a GWAS approach. Sample sizes ranged from 44 to 883 participants, with a median sample size of approximately 215.

### 3.3 Genes and variants

Table 1 shows a summary of all included studies. The most frequently studied genes were from the complement pathway (CFH, C3, C2, CFB, CFI), AMD-susceptibility loci (ARMS2, HTRA1), angiogenesis (VEGFA), and oxidative stress (GSTM1, GSTT1, GSTP1). Genotyping methods included PCR-RFLP, real-time PCR, Sanger sequencing, TaqMan assays, multiplex PCR, and mass spectrometry (iPLEX MALDI-TOF). The number of polymorphisms investigated per study ranged from 1 to 12, and one study employed a GWAS approach.

The CFH gene emerged as the most consistently replicated AMD susceptibility locus. The Y402H variant (rs1061170) demonstrated significant associations with AMD risk across multiple populations (Egyptian: OR = 1.762–17.336, p = 0.00214; Iranian: OR = 1.81–4.83, p < 0.001–0.002; Israeli: OR = 1.3–2.6, p = 0.0002; Turkish: OR = 0.3–4.88, p = 0.0001–0.011).

**FIGURE 3:**
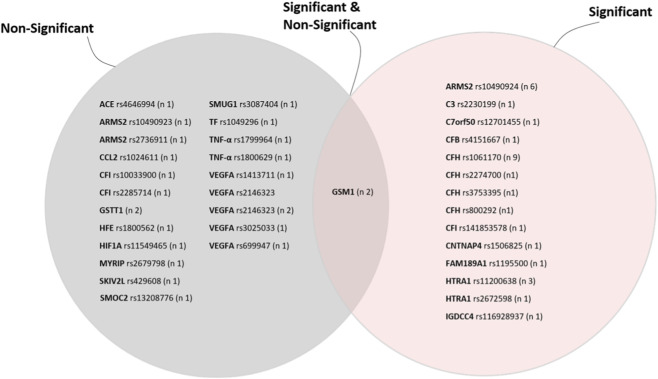
The diagram summarizes SNPs reported across Middle Eastern studies, categorized as significantly associated with AMD (right circle), not significantly associated (left circle), and SNPs found as both significant and non-significant across different studies (overlapping region). Variants without reported p-values were not included. n; number of included studies reporting the variants.

The ARMS2 A69S variant (rs10490924) and HTRA1 promoter variant (rs11200638) showed robust associations with AMD. Israeli studies reported ORs of 3.1 (95% CI: 2.2-4.5, p<0.0001) and 2.7 (95% CI: 1.9-4.0, p<0.0001) respectively. Turkish studies consistently confirmed the association of ARMS2 A69S variant with AMD risk, with odds ratios ranging from 3.0 to 8.56 (95% CI: 1.23–20.83) and p values ≤ 0.008, including highly significant results (p < 0.0001). Other complement genes (C2, C3, CFB, CFI) showed inconsistent replication. A Turkish study found GSTM1 null genotype associated with increased AMD risk (OR: 1.82, p = 0.01). The studied variants in the following genes showed no significant association with AMD in studied Middle Eastern populations: ACE, CCL2, HFE, HIF1A, MYRIP, SKIV2L, SMOC2, SMUG1, TF, TNF-α, and VEGF. Figure 3 shows a Venn diagram summarizing SNPs significantly associated with AMD, not associated, and those reported in both categories across Middle Eastern studies.

## 4 Discussion

A major limitation of AMD genetic research in the Middle East is the near-absence of large genotype-phenotype studies. Importantly, the findings of this systematic review are based on a limited and geographically unbalanced set of studies from Middle Eastern populations. With the exception of a single GWAS study, all identified studies relied on candidate-gene approaches that targeted variants identified in other populations. This approach substantially limits the discovery of population-specific or rare risk variants shaped by the region’s unique demographic history, complex admixture patterns, and relatively high rates of consanguinity. As a result, Middle Eastern-specific AMD susceptibility loci, or low-frequency variants may remain undetected. This limitation is further compounded by the small number of analyzed variants. This is despite the identification of more than 50 AMD-associated loci in European and East Asian populations (Fritsche et al., 2013; Gorman et al., 2022; Han et al., 2020; He et al., 2024; Klein et al., 2005; Kvale et al., 2015; Winkler et al., 2020).

Identified Middle Eastern studies examined a limited number of genetic variants, with most assessing only one to four polymorphisms (25 studies) and only two studies evaluating up to 12 variants per study. Consequently, the contribution of many established genetic variants remains uncovered. Moreover, even for well-studied loci such as CFH and ARMS2/HTRA1, analyses were often limited to one or a few variants, precluding assessment of haplotypes or independent association signals.

Despite these limitations, CFH, ARMS2, and HTRA1 were among the most frequently reported loci associated with AMD susceptibility in studied Middle Eastern populations. The CFH Y402H variant (rs1061170) showed generally positive but heterogeneous associations with AMD in studied Middle Eastern populations, with effect sizes comparable to those observed in other populations (odds ratios extending to approximately five) (Klein et al., 2005; Zareparsi et al., 2005; Haines et al., 2005). This missense variant results in a tyrosine-to-histidine substitution at amino acid position 402 within the complement control protein domain of the CFH protein. The cross-population consistency of this association is biologically plausible, given that the Y402H substitution impairs complement regulation at the retinal surface, a central mechanism in AMD pathogenesis (Skerka et al., 2007; Clark et al., 2010; Wilke and Apte, 2024). Individuals carrying one copy of the risk allele (T) have approximately 2.5-fold increased odds of developing AMD, while homozygotes exhibit 6-fold increased risk compared to non-carriers (Thakkinstian et al., 2006). The variant has been associated with both dry and wet forms of AMD. Functional study showed that Y402H CFH exhibits reduced binding to oxidized lipids (like malondialdehyde epitopes), impairing protection of retinal cells from damage (Weismann et al., 2011).

Additional CFH polymorphisms have been implicated, suggesting that multiple regulatory and coding variants may modulate complement activity. CFH rs800292 is a coding variant located in exon 2 of the CFH gene, resulting in an isoleucine-to-valine substitution at amino acid position 62. The variant is located within the first complement control protein domain of CFH and influence the protein’s binding affinity to C3b and its cofactor activity in the alternative complement pathway (Tortajada et al., 2009). The CFH rs2274700 is a synonymous variant located in exon 10 and may function by altering CFH expression levels and those of other complement factor-related genes (Francis et al., 2007). The CFH rs3753395 is a non-coding variant located in an intronic region and showed a strong association with CFH mRNA expression (Zhang et al., 2014).

In addition to CFH, downstream components of the complement cascade have also been examined, further supporting complement dysregulation as a central pathogenic mechanism in AMD. C3 is the central component of all complement pathways and is essential for innate immunity and inflammatory responses (Janssen and Gros, 2007). The C3 rs2230199 variant is a non-synonymous coding change that results in an arginine-to-glycine substitution at position 102 of the C3 protein. This variant has demonstrated functional effects on C3, including altered binding to monocyte complement receptors. The glycine allele has been associated with IgA nephropathy, systemic vasculitis, partial lipodystrophy, and membranoproliferative glomerulonephritis type II (Spencer et al., 2008).

Rare variants affecting complement regulation have also been reported, highlighting the possible contribution of highly penetrant mutations alongside common risk alleles. CFI rs141853578 is a rare missense variant (rs141853578) resulting in a glycine-to-arginine substitution at amino acid position 119 of the protein. CFI is a serine protease that serves as a critical regulator of the complement system by cleaving C3b and C4b. The variant impairs C3b degradation and shows reduced expression and activity compared with wild type (Geerlings et al., 2018; Van De Ven et al., 2013). The CFB rs4151667 is a missense variant resulting in a leucine-to-histidine substitution at amino acid position 9 of the protein. A meta-analysis demonstrated that the CFB rs4151667 variant is associated with a protective effect against AMD (Thakkinstian et al., 2012).

Beyond the complement pathway, associations have been reported across multiple studies at the chromosome 10q26 locus, a major non-complement genetic determinant of AMD. ARMS2 is located on chromosome 10q26 near HTRA1, and the two loci exhibit strong linkage disequilibrium, making it challenging to distinguish their independent contributions to AMD risk. Functional studies suggest that ARMS2 variants may influence mitochondrial homeostasis in the retina (Fritsche et al., 2008). In this study cohorts, ARMS2 rs10490924 showed an odds ratio of approximately 3.0 for wet AMD. The rs10490924 is a non-synonymous coding variant that represents another common risk factor for AMD. This variant results in an alanine-to-serine substitution at position 69 of the ARMS2 protein, a 107-amino acid protein of unknown function that localizes to the mitochondria (Kanda et al., 2007). The rs10490924 risk allele (TT) confers odds ratios of 10.99 (Chen et al., 2011).

Regulatory variants at HTRA1 have also been extensively investigated as a potential driver of AMD risk. HTRA1 rs11200638 is a promoter variant located 512 base pairs upstream of the HTRA1 transcription start site. This regulatory variant upregulates HTRA1 expression in retinal tissues. HTRA1 encodes a secreted serine protease that drives extracellular matrix degradation, which may lead to disruption of Bruch’s membrane integrity and progression of choroidal neovascularization (Yang et al., 2006). The rs11200638 risk haplotype (A) enhances inflammatory responsiveness of the HtrA1 promoter, leading to increased HtrA1 expression. Elevated HtrA1 levels showed to induce apoptosis and growth inhibition in Cultured adult retinal pigment epithelial cells, supporting a pathogenic role in AMD (He et al., 2022).

In contrast to complement and 10q26 loci, evidence for genes involved in oxidative stress pathways has been less consistent across Middle Eastern studies. A Turkish study (Güven et al., 2011) reported an association between the GSTM1 null genotype and increased AMD risk, particularly for dry AMD, whereas an Iranian study (Othman et al., 2012) found no such association. These discrepancies underscore the difficulty of reliably assessing genes with modest effects in underpowered studies and highlight the need for standardized phenotyping and larger, multi-center investigations. The GSTM1 gene is known for its role in detoxifying harmful substances and protecting cells from oxidative stress. Individuals with the GSTM1 null genotype may exhibit altered antioxidant defenses, thereby increasing their susceptibility to oxidative damage in retinal cells. This increased vulnerability to oxidative stress may contribute to the progression of AMD, as the cumulative effects of oxidative damage can lead to retinal cell degeneration and impaired visual function. Furthermore, studies have indicated that individuals with the GSTM1 null genotype may experience an earlier onset of AMD symptoms, potentially due to the diminished capacity for detoxifying reactive oxygen species that are prevalent in the aging retina (Chen and Luo, 2022).

Taken together, the available evidence from Middle Eastern populations highlights several key patterns beyond individual gene associations. First, there is substantial phenotype heterogeneity, with inconsistent classification of AMD subtypes across studies, which limits comparability and interpretation of genetic effects. Second, variant coverage remains limited, as most studies investigated a small number of preselected polymorphisms rather than performing comprehensive genomic analyses. Third, the evidence base is heavily skewed toward a few populations, further restricting regional inference. These factors collectively underscore that current findings reflect partial and potentially biased representations of the genetic architecture of AMD in the Middle East. Future research should prioritize standardized phenotyping using harmonized clinical criteria for AMD subtypes to improve comparability across studies. Broader variant interrogation through genome-wide approaches, including GWAS and whole-exome or whole-genome sequencing, is needed to capture both common and rare variants that may be specific to Middle Eastern populations. In addition, establishing multi-center, regionally coordinated studies will be essential to increase sample sizes, reduce geographic bias, and enable more representative and statistically robust analyses.

This review is limited by the small number of studies, modest sample sizes, and strong geographic imbalance across the Middle East, with available data derived from a limited subset of countries. This marked geographic imbalance, with the majority of studies originating from Iran and Turkey, substantially limits the generalizability of these findings to the broader Middle Eastern region, particularly given the known genetic diversity and underrepresentation of many populations in this region (Ramaswamy et al., 2022). Additional limitations include the predominance of candidate-gene studies and heterogeneity in phenotype reporting and genotyping methods. An additional limitation is that the review was not prospectively registered in PROSPERO, which may increase the risk of reporting bias.

## 5 Conclusion

This systematic review demonstrates that CFH, ARMS2, and HTRA1 variants are among the most commonly reported genetic risk factors for AMD in the studied Middle Eastern populations, with effect sizes broadly consistent with those reported in European and Asian cohorts. However, the overall evidence base is strikingly limited: only 28 studies encompassing 7,319 participants represent the entirety of published AMD genetic research across a region of more than ∼500+ million people spanning 17 countries (The Middle East Population, 2025). The available data are heavily skewed geographically, with most studies originating from a small number of countries and 11 Middle Eastern nations lacking AMD genetic research. Notably, the high prevalence of consanguineous marriage in many Middle Eastern populations offers a unique opportunity to uncover recessive or population-specific AMD susceptibility loci that are hard to detect in outbred populations. Addressing these gaps will require large-scale GWAS, genome sequencing, and multi-center regional consortia, alongside integration with global genomics initiatives. This combined approach will enable comprehensive risk assessment and the identification of novel, population-relevant therapeutic targets.

## Data Availability

The original contributions presented in the study are included in the article/Supplementary Material, further inquiries can be directed to the corresponding author.
