# Correction: Genetic determinants of age-related macular degeneration in Middle Eastern populations: a systematic review

**DOI:** 10.3389/fgene.2026.1893561

**Published:** 2026-07-08

**Authors:** Naif S. Sannan

**Affiliations:** 1 Department of Clinical Laboratory Sciences, College of Applied Medical Sciences, King Saud bin Abdulaziz University for Health Sciences, Jeddah, Saudi Arabia; 2 Biomedical Research Department, King Abdullah International Medical Research Center, Jeddah, Saudi Arabia

**Keywords:** age-related macular degeneration, AMD, ARMS2, CFH, genetics, HTRA1, middle east, polymorphism

There was a mistake in Table 1 as published. The reference for Furundaoturan et al. (2025) was erroneously written as Grunin et al. (2024). It should be Furundaoturan et al. (2025). In addition, the reference for Grunin et al. (2024) was erroneously written as Furundaoturan et al. (2025). It should be Grunin et al. (2024).

The original article has been updated.

