# Supplementary material for: Genetic determinants of age-related macular degeneration in Middle Eastern populations: a systematic review
Source: Front Genet. 2026 May 8;17:1776779. doi: 10.3389/fgene.2026.1776779 (PMC13193682; doi:10.3389/fgene.2026.1776779)
Supplement: Supplementary file 2 [file Table1.docx]

**Supplementary Table 1.** Search Strategy for Genetic Studies of Age-Related Macular Degeneration in Middle Eastern Populations

| **Database** | **Search Terms / Strategy** |
| --- | --- |
| **PubMed** | ("Macular degeneration"[MeSH Terms] OR "drusen" OR "geographic atrophy" OR "AMD"[Title/Abstract] OR "Macula"[Title/Abstract] OR "Macular"[Title/Abstract] OR "Macular Degeneration"[Title/Abstract])  AND  ("Gene"[Title/Abstract] OR "Genes"[Title/Abstract] OR "Genome"[Title/Abstract] OR "Genetic"[Title/Abstract] OR "Genetics"[Title/Abstract] OR "Genetics"[MeSH Terms] OR "Polymorphism"[Title/Abstract] OR "Polymorphisms"[Title/Abstract] OR "Variant"[Title/Abstract] OR "Variants"[Title/Abstract] OR "SNP"[Title/Abstract] OR "SNPs"[Title/Abstract] OR "Mutation"[Title/Abstract] OR "Mutations"[Title/Abstract] OR "Genotype"[Title/Abstract] OR "Genotyping"[Title/Abstract] OR "Sequencing"[Title/Abstract] OR "GWAS"[Title/Abstract] OR "Allele"[Title/Abstract] OR "Alleles"[Title/Abstract] OR "CFH"[Title/Abstract] OR "ARMS2"[Title/Abstract] OR "HTRA1"[Title/Abstract] OR "C3"[Title/Abstract] OR "C2"[Title/Abstract] OR "CFB"[Title/Abstract] OR "CFI"[Title/Abstract] OR "C9"[Title/Abstract] OR "VEGFA"[Title/Abstract] OR "Complement factor"[Title/Abstract])  AND  ("Middle Eastern people"[MeSH Terms] OR "Middle East" OR "Middle Eastern"[Title/Abstract] OR "Arab"[Title/Abstract] OR "Persian"[Title/Abstract] OR "Gulf"[Title/Abstract] OR "MENA"[Title/Abstract] OR "Saudi Arabia"[Title/Abstract] OR "United Arab Emirates"[Title/Abstract] OR "Qatar"[Title/Abstract] OR "Kuwait"[Title/Abstract] OR "Bahrain"[Title/Abstract] OR "Oman"[Title/Abstract] OR "Yemen"[Title/Abstract] OR "Iraq"[Title/Abstract] OR "Syria"[Title/Abstract] OR "Jordan"[Title/Abstract] OR "Lebanon"[Title/Abstract] OR "Cyprus"[Title/Abstract] OR "Palestine"[Title/Abstract] OR "Egypt"[Title/Abstract] OR "Turkey"[Title/Abstract] OR "Iran"[Title/Abstract] OR "Israel"[Title/Abstract] OR "Saudi"[Title/Abstract] OR "Jordanian"[Title/Abstract] OR "Qatari"[Title/Abstract] OR "Kuwaiti"[Title/Abstract] OR "Bahraini"[Title/Abstract] OR "Omani"[Title/Abstract] OR "Yemeni"[Title/Abstract] OR "Iraqi"[Title/Abstract] OR "Syrian"[Title/Abstract] OR "Emirati"[Title/Abstract] OR "Lebanese"[Title/Abstract] OR "Cypriot"[Title/Abstract] OR "Palestinian"[Title/Abstract] OR "Egyptian"[Title/Abstract] OR "Turkish"[Title/Abstract] OR "Iranian"[Title/Abstract] OR "Israeli"[Title/Abstract]) |
| **Web of Science core collection** | TS=( "degeneration, macular" OR "macular degenerations" OR "macular dystrophy" OR "dystrophy, macular" OR "macular dystrophies" OR "maculopathy" OR "maculopathies" OR "age-related macular degeneration" OR "age related macular degeneration" OR "age-related macular degenerations" OR "age-related maculopathies" OR "age related maculopathies" OR "age-related maculopathy" OR "age related maculopathy" OR "maculopathies, age-related" OR "maculopathy, age-related" OR "maculopathy, age related" OR "macular degeneration, age-related" OR "macular degeneration, age related" OR "AMD" OR "drusen" OR "geographic atrophy" )  AND  TS=( "gene" OR "genes" OR "genome" OR "genetic" OR "genetics" OR "polymorphism" OR "polymorphisms" OR "variant" OR "variants" OR "SNP" OR "SNPs" OR "mutation" OR "mutations" OR "genotype" OR "genotyping" OR "sequencing" OR "GWAS" OR "allele" OR "alleles" OR "CFH" OR "ARMS2" OR "HTRA1" OR "C3" OR "C2" OR "CFB" OR "CFI" OR "C9" OR "VEGFA" OR "complement factor" )  AND  TS=( "Middle East" OR "Middle Eastern" OR "Middle Eastern people" OR "People, Middle Eastern" OR "Middle Easterners" OR "Middle Easterner" OR "Near Easterners" OR "Near Easterner" OR "Southwestern Asians" OR "Asian, Southwestern" OR "Southwestern Asian Person" OR "Asian Person, Southwestern" OR "Person, Southwestern Asian" OR "Southwestern Asian Persons" OR "Southwestern Asian People" OR "Asian People, Southwestern" OR "Asian Peoples, Southwestern" OR "People, Southwestern Asian" OR "Peoples, Southwestern Asian" OR "Southwestern Asian Peoples" OR "Middle Eastern Person" OR "Middle Eastern Persons" OR "Person, Middle Eastern" OR "Arab" OR "Persian" OR "Gulf" OR "MENA" OR "Saudi Arabia" OR "UAE" OR "Qatar" OR "Kuwait" OR "Bahrain" OR "Oman" OR "Yemen" OR "Iraq" OR "Syria" OR "Jordan" OR "Lebanon" OR "Cyprus" OR "Palestine" OR "Egypt" OR "Turkey" OR "Iran" OR "Israel" OR "Saudi" OR "Jordanian" OR "Qatari" OR "Kuwaiti" OR "Bahraini" OR "Omani" OR "Yemeni" OR "Iraqi" OR "Syrian" OR "Emirati" OR "Lebanese" OR "Cypriot" OR "Palestinian" OR "Egyptian" OR "Turkish" OR "Iranian" OR "Israeli" ) |
| **Medline** | TS=( "degeneration, macular" OR "macular degenerations" OR "macular dystrophy" OR "dystrophy, macular" OR "macular dystrophies" OR "maculopathy" OR "maculopathies" OR "age-related macular degeneration" OR "age related macular degeneration" OR "age-related macular degenerations" OR "age-related maculopathies" OR "age related maculopathies" OR "age-related maculopathy" OR "age related maculopathy" OR "maculopathies, age-related" OR "maculopathy, age-related" OR "maculopathy, age related" OR "macular degeneration, age-related" OR "macular degeneration, age related" OR "AMD" OR "drusen" OR "geographic atrophy" )  AND  TS=( "gene" OR "genes" OR "genome" OR "genetic" OR "genetics" OR "polymorphism" OR "polymorphisms" OR "variant" OR "variants" OR "SNP" OR "SNPs" OR "mutation" OR "mutations" OR "genotype" OR "genotyping" OR "sequencing" OR "GWAS" OR "allele" OR "alleles" OR "CFH" OR "ARMS2" OR "HTRA1" OR "C3" OR "C2" OR "CFB" OR "CFI" OR "C9" OR "VEGFA" OR "complement factor" )  AND  TS=( "Middle East" OR "Middle Eastern" OR "Middle Eastern people" OR "People, Middle Eastern" OR "Middle Easterners" OR "Middle Easterner" OR "Near Easterners" OR "Near Easterner" OR "Southwestern Asians" OR "Asian, Southwestern" OR "Southwestern Asian Person" OR "Asian Person, Southwestern" OR "Person, Southwestern Asian" OR "Southwestern Asian Persons" OR "Southwestern Asian People" OR "Asian People, Southwestern" OR "Asian Peoples, Southwestern" OR "People, Southwestern Asian" OR "Peoples, Southwestern Asian" OR "Southwestern Asian Peoples" OR "Middle Eastern Person" OR "Middle Eastern Persons" OR "Person, Middle Eastern" OR "Arab" OR "Persian" OR "Gulf" OR "MENA" OR "Saudi Arabia" OR "UAE" OR "Qatar" OR "Kuwait" OR "Bahrain" OR "Oman" OR "Yemen" OR "Iraq" OR "Syria" OR "Jordan" OR "Lebanon" OR "Cyprus" OR "Palestine" OR "Egypt" OR "Turkey" OR "Iran" OR "Israel" OR "Saudi" OR "Jordanian" OR "Qatari" OR "Kuwaiti" OR "Bahraini" OR "Omani" OR "Yemeni" OR "Iraqi" OR "Syrian" OR "Emirati" OR "Lebanese" OR "Cypriot" OR "Palestinian" OR "Egyptian" OR "Turkish" OR "Iranian" OR "Israeli" ) |
